# Introducing, OncoTarget

**Published:** 2010-05-01

**Affiliations:** Roswell Park Cancer Inst., Buffalo, NY; Roswell Park Cancer Inst., Buffalo, NY

Previous decades have witnessed intensive accumulation of knowledge on oncogenes, oncoproteins, tumor suppressors, signal transduction, cellular processes (such as proliferation, differentiation, apoptosis, autophagy, migration), cellular responses (such as DNA damage, heat shock responses), intercellular interactions (cell-cell communications), and the function and dysfunction of cells and organ systems. Therapeutic targeting of these molecules and processes is becoming the most rapidly growing area in cancer research and the field is becoming increasingly complex and multidisciplinary. Knowledge must be translated from basic research to the clinic and vice versa, encompassing the varied fields of cell and molecular biology, pharmacology, pathology and physiology, oncology and other medical disciplines.

Unexpectedly, in different medical disciplines, similar targets for therapy have been identified. Therefore, agents developed for specific diseases might be useful in treating cancer and vice versa. Some drugs that are used to treat infections and other disorders impact cancer-relevant targets. Some such drugs are particularly attractive as candidate anti-cancer agents since they have already been approved for clinical use and their effects and side effects are known. For example, anti-malarial and anti-epileptic drugs (and even teratogens) are being considered as anti-cancer drugs. On the other hand, some immunosuppressants and anti-cancer drugs are now indicated for therapy of atherosclerosis, neurodegeneration and even aging. Furthermore, the term target includes not only molecules (such as p53), but also cells (e.g., endothelial cells are targets in oncology, ophthalmology and cardiology), cellular functions and responses (e.g., cell cycle progression, heat shock response, etc.) and even as physiological features such as hypoxia (e.g., exploitation of tumor hypoxia for its destruction by anaerobic bacteria). Moreover, many of these targets might be relevant not only as therapeutic targets, but also diagnostic targets. Here, we introduce the term “oncotarget” as a descriptor that would encompass all molecules, pathways, cellular functions, cell types and even tissues that can be viewed as targets relevant to cancer and other diseases as well.

The word “oncogene” retrieves 168,147 papers from PubMed, starting from 1969 (Huebner et al). The word “oncoprotein” retrieves 157,259 papers, starting from with one published in 1970 (Ting et al). The word “target” retrieves a half million papers. In contrast, searching with the word “oncotarget” retrieves no real hits from the current PubMed database (only several papers with “oncotarget” as part of an e-mail address).

The time is right for a new term, a new concept and a new journal, all specifically focused on the field of cancer-relevant targets, while also broadly encompassing the complexity of the field. In several inaugural issues of Oncotarget, there are papers on basic, translational and clinical research, paradigm-shifting articles and systematic reviews, opinions and perspectives. In addition to its simultaneously unique and broad scope, Oncotarget offers other unique features (see Homepage) that will make it a high impact journal for the twenty-first century.

